# Novel microwave assisted carboxymethyl-graphene oxide and its hepatoprotective activity

**DOI:** 10.1186/s40360-024-00768-0

**Published:** 2024-08-13

**Authors:** Hebat-Allah S. Tohamy, Fatma El-Zahraa S. Mohamed, Mohamed El-Sakhawy

**Affiliations:** 1 Cellulose and Paper Department, National Research Centre, 33 El Bohouth Str., P.O. 12622, Dokki, Giza, Egypt; 2 Nutrition and Food Science Department, Research of food Industries and Nutrition Institute, National Research Centre, 33 El Bohouth St. (former El Tahrir St.), P.O. 12622, Dokki, Cairo, Egypt

**Keywords:** Graphene oxide, Carbon tetracholoride (CCL_4_), Biochemical analysis, Histopathological liver

## Abstract

This study reports a novel, eco-friendly; fast and cost-effective microwave method for synthesizing carboxymethylated graphene oxide (CMGO) from sugarcane residues. Fourier-transform infrared spectroscopy (FTIR) confirmed successful CMGO synthesis through the presence of characteristic peaks at 1567.93 and 1639.29 cm^−1^ (COONa vibrations) and increased CH_2_ intensity compared to unmodified graphene oxide (GO). Furthermore, CMGO derived from sugarcane residues demonstrated potential in mitigating the side effects of toxic materials like carbon tetrachloride (CCl_4_). Treatment with CMGO partially reduced elevated levels of liver enzymes (ALT and AST) and nitrogenous waste products (urea and uric acid) in CCl_4_-induced liver damage models, suggesting an improvement in liver function despite ongoing cellular damage.This work paves the way for a sustainable and economical approach to produce functionalized graphene oxide with promising biomedical applications in alleviating toxin-induced liver injury.

**Graphical abstract:**

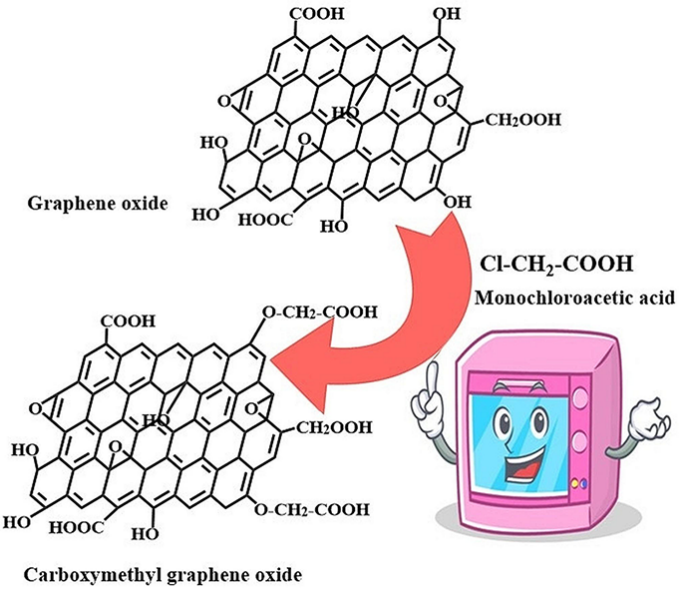

## Introduction

The fact that millions of people worldwide suffer from liver illness, which is made worse by things like alcoholism, smoking, and environmental pollution, emphasizes how important the liver is to the detoxification process [1–4]. In order to promote liver health, researchers use controlled models that use carbon tetrachloride (CCl_4_) to produce liver injury. This allows them to better understand the mechanisms underlying liver damage and develop protective methods [1, 5]. Imagine the liver as a shield, keeping dangerous toxins away from our important organs. However, barriers can also be overpowered. Disease results from the harm done to liver cells when it occurs, akin to a castle being invaded. Even with the body’s built-in detoxifying system, liver disorders can pose a serious threat to public health. Scientists are working hard to develop novel treatments that will fortify this vital barrier, promote liver function, and potentially repair damaged cells [6]. At first appearance, carbon tetrachloride (CCl_4_), a transparent and versatile liquid, can appear harmless. Degreasers, refrigerants, and even fire extinguisher fluids are contained within. But take caution—this seemingly useful instrument has a hidden edge that is capable of doing significant damage [7].

The remarkable properties of graphene (G) and its nanostructures make them game-changers in medical and engineering fields [8]. One of the most prominent properties of graphene oxide (GO), a derivative of graphene (G), is its regulated hydrophilicity. Because of these characteristics and the help of surface hydroxyl groups, GO is more practical than the original G. Moreover, its minuscule dimensions and vast surface area offer intriguing opportunities for an array of uses [9–11]. Modification of GO by carboxymethylation is one of the best methods. Through this process, some of the hydroxyl groups in the GO are swapped out for carboxymethyl groups, which increase the hydrophilicity of the material. Previously, carboxymethylation was achieved by reacting with monochloro acetic acid in severe environments [9, 10, 12]. In this work, we will use the microwave method to prepare carboxymethylated GO (CMGO) in a few minutes instead of the conventional method which was taken many hours. This technology is original and novel in that it attaches carboxymethyl groups to GO utilizing an ecologically friendly microwave process. We shall then look into how this changed GO affects liver protection. In a previous study, the protective effect of date fruit extract against toxicity in male rats was investigated [13]. In this study, sugar cane juice was introduced for rats feeding to study its protection against CCl_4_ toxicity.

## Materials and methods

### Materials

The study made use of easily accessible materials for the liver injury model as well as food ingredients. Using sugarcane bagasse (SC) from Quena Company for Paper Industry in Egypt, carboxymethylated graphene oxide (CMGO) was produced as a basis. Carbon tetrachloride (CCL4), available orally from BDH chemicals Ltd (Poole, England), was used to create controlled liver damage in animal models.

Ingredients in the diet were carefully selected to ensure the best possible nutrition and experimental control. The protein source was casein (Al-Ahram Laboratory Chemicals, Egypt), and the dietary fiber was cellulose (Laboratory of Rasayan, Fine Chemical Limited, Mumbai, India). Dietary balance was guaranteed by vital salts and vitamins from BDH (England) and Fluka (Germany), respectively. The animals’ food requirements were supplied by additional purchases from the local market.

Accurate evaluation of liver and renal function was assured by trustworthy diagnostic instruments. Key markers such as ALT, AST, urea, creatinine, and uric acid might be determined spectrophotometrically with the help of Diamond diagnostics kits (MDSS GmbH, Hannover, Germany). Every chemical utilized was analytical grade, and it wasn’t further purified before usage.

### Preparation of graphene oxide from SC

0.5 g SC and 100 mg ferrocene were placed into a muffle furnace at 300 °C for 10 min to attain a black powdered GO [9].

### Preparation of carboxymethyl-GO (CMGO)

The prepared GO (15 g) was mixed with isopropanol (400 ml), 30% NaOH solution and monochloro acetic acid (18 g), followed by heating in the microwave until complete dissolution, as illustrated in the graphical scheme. The prepared CMGO was precipitated with 70% methanol, filtered, and dried at oven.

#### Characterization

##### FTIR spectra

FTIR spectroscopy which was recorded by Mattson-5000 (Unicam, Somerset, United Kingdom) employing the KBr disk method across the wavenumber range of 4000–1000 cm^−1^.

##### XRD spectra

XRD determined by Bruker D8 Advance X-ray diffractometer (Karlsruhe, Germany) using copper (K*α*) radiation (1.5406 Å) at a 40 kV voltage and a 40 mA current.

Both of crystallinity index Cr.I. (%) was determined using Eq. (1).

1 $${\text{Cr}}{\text{.I}}{\text{.}}\,\left( \% \right)\,=\,\left( {{{\text{A}}_{\text{C}}}/{{\text{A}}_{\text{t}}}} \right)\, \times \,100$$

where A_C_ is the crystalline peak area and A_t_ is the total area [9, 14].

### Transmission electron microscope (TEM) analysis

TEM images were taken with a JEOL JEM-2100 electron microscopy at an acceleration voltage of 120 kV.

### Animals

Twenty-six male albino rats were removed from the animal housing of the National Research Centre (NRC, Dokki, Egypt). The distribution of weight was 200 ± 150 g. The animals housed in individual rooms were observed by the facility’s vets, who also carried out their own independent assessments. This experiment was carried out in compliance with Ain Shams University’s ethical research guidelines using the [RE (223) 23] Code Number in the Experimental Animal Research Unit. The animals were housed in separate rooms within the facility, under the constant supervision of a veterinarian who carried out unbiased examinations.

### Experimental diet

The basal diet was carried out in accordance with AIN- 93 [15]. Before being taken orally at a dose of 0.4 mg per day, the CMGO was ground into a fine powder and diluted 1 g in 10 ml water and 10 ml sugar cane juice. The rats were adapted for two weeks before the trial began. Water was provided ad libitum. Rats were separated into four groups and fed the following diets for 45 days: Group 1 (8 rats) was the control negative group (basal diet), Group 2 (8 rats) was the control positive group (basal diet + 0.4 mg CCl_4_ orally), Group 3 (8 rats) was the control positive group (basal diet + 0.4 mg CCl_4_ orally + 1 g CMGO dissolved in 10 ml water), and Group 4 (8 rats) was the control positive group + (basal diet + 0.4 mg CCl_4_ orally + 1 g CMGO dissolved in 10 ml SC juice. During the experiment period, the quantities of diet, which were consumed and/ or wasted, were recorded every day.

### Biological study

Rat’s weight was recorded weekly, to determine Food Intake and Body Weight Gain % according to Chapman et al. [16]. At the end of the twenty-two-day trial, rats were slain after an overnight fast under halothane anaesthesia. A tiny part of the blood was taken from the hepatic portal vein, and the remainder was allowed to clot at room temperature for 15 min before being centrifuged at 3000 rpm for 20 min. Serum was properly separated and stored at −20 °C in clean, securely fitting plastic tubes until analysis.

### Liver and kidney functions

Using a colorimetric endpoint approach, the measurements of Alanine Aminotransferase (ALT) and Aspartate aminotransferase (AST) were carried out in accordance with the instructions supplied by Diamond Diagnostics, MDSS GmbH, Hannover, Germany. Urea and creatinine were measured in accordance with the procedures at Diamond Diagnostics, MDSS GmbH, Hannover, Germany.

### Histopathological examination

After the rats in each group were killed, samples of the spleen, liver, and thymus were taken. Bancroft and Gamble (2008) states that after the tissues were cleansed with a normal saline solution to remove any blood, they were fixed with 10% neutral formalin. After that, they were taken to the Faculty of Veterinary Medicine at Cairo University to undergo histological examination [17].

### Statistical analysis

With the aid of automated SPSS software (SAS Institute, Cary, NC), the data was statistically assessed. A one-way ANOVA (Analysis of Variance) test with Duncan’s multiple range tests and p significance between different groups was used to assess the impact of various therapies [18].

## Results

### FTIR spectra

**Fig. 1:**
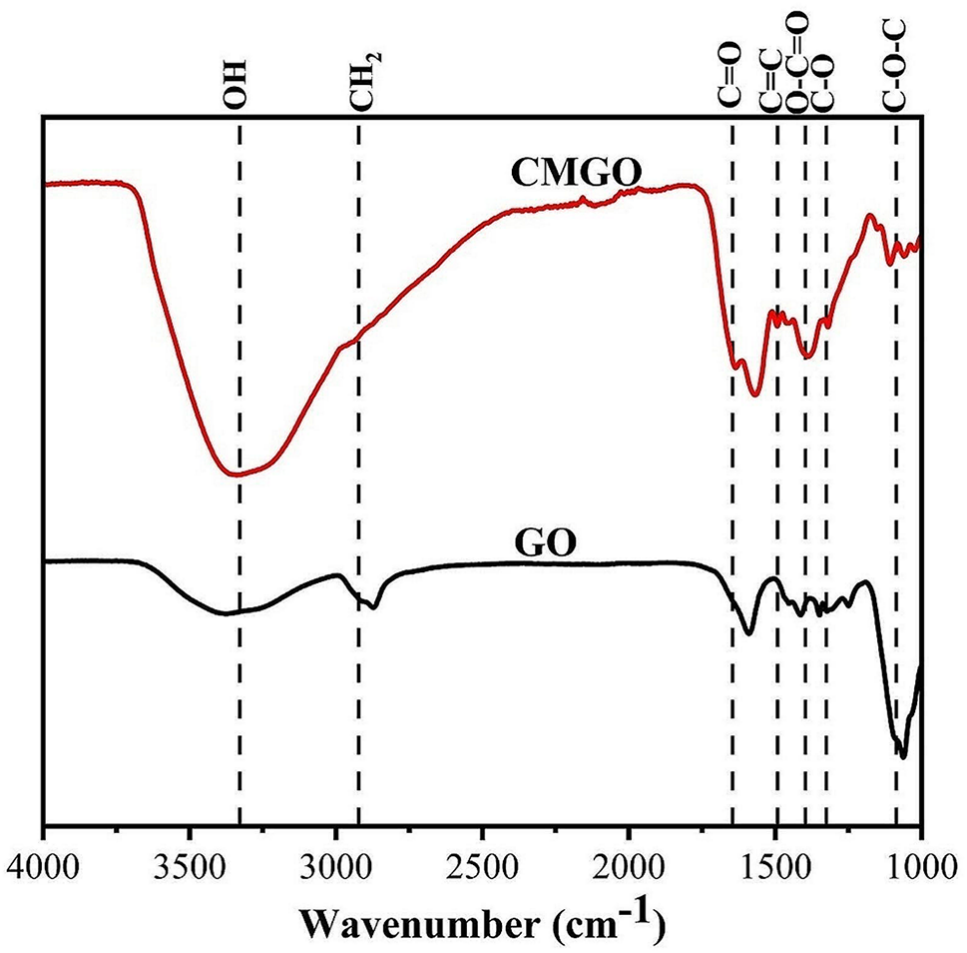
FTIR spectra of GO and CMGO

Figure 1 shows the FTIR spectra for GO and CMGO with peaks between 3353.80 and 3384.81 (O–H), 2910.36–2950.73 (CH_2_), 1595.01–1639.29 (C = O), 1461.93–1486.93 (C = C), 1350.06–1390.50 (O–C = O), 1319.15–1321.13 (C–O), 1064.62–1110.86 (C–O–C) cm^−1^ [9, 10].

**Table 1:** MHBS and LOI of GO and CMGO

Groups	MHBS	LOI
GO	1.38	0.83
CMGO	0.73	0.25

For CMGO the C = O peak was splitted to 1567.93 and 1639.29 cm^−1^ related to anti-symmetric and symmetric vibrations of COONa [9, 19]. The relative intensity of CH_2_ group for CMGO (i.e. 1.12) was increased compared to GO (i.e. 0.60) which prove the carboxymethylation process. Moreover the Means hydrogen bond strength (MHBS) and Lateral order index (LOI, values are indicative of the cellulose crystallinity) for CMGO, as shown in Table 1, are lower than GO which means the decreasing of H–bonding due to carboxymethylation [20, 21].

### XRD spectra

**Fig. 2:**
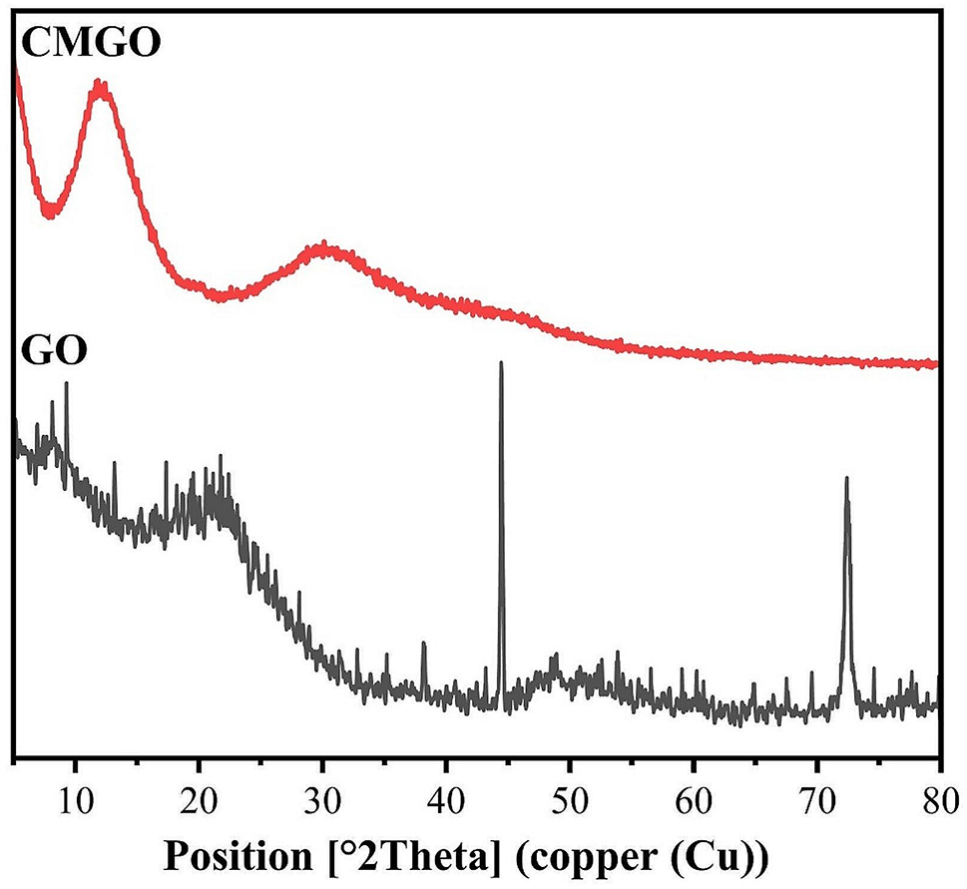
XRD spectra of GO and CMGO

Figure 2 shows the XRD spectra of GO and CMGO. The GO shows reflections at 8.19 and 21.74º correlated to (001) and (002) plans [9, 11]. The CMGO showed broadening of the peaks at 11.69 and 29.47º indicates the amorphous structure of CMGO [20, 22]. The calculated Cr.I was 41.75 and 32.02% for GO and CMGO, respectively.

### TEM analysis

**Fig. 3:**
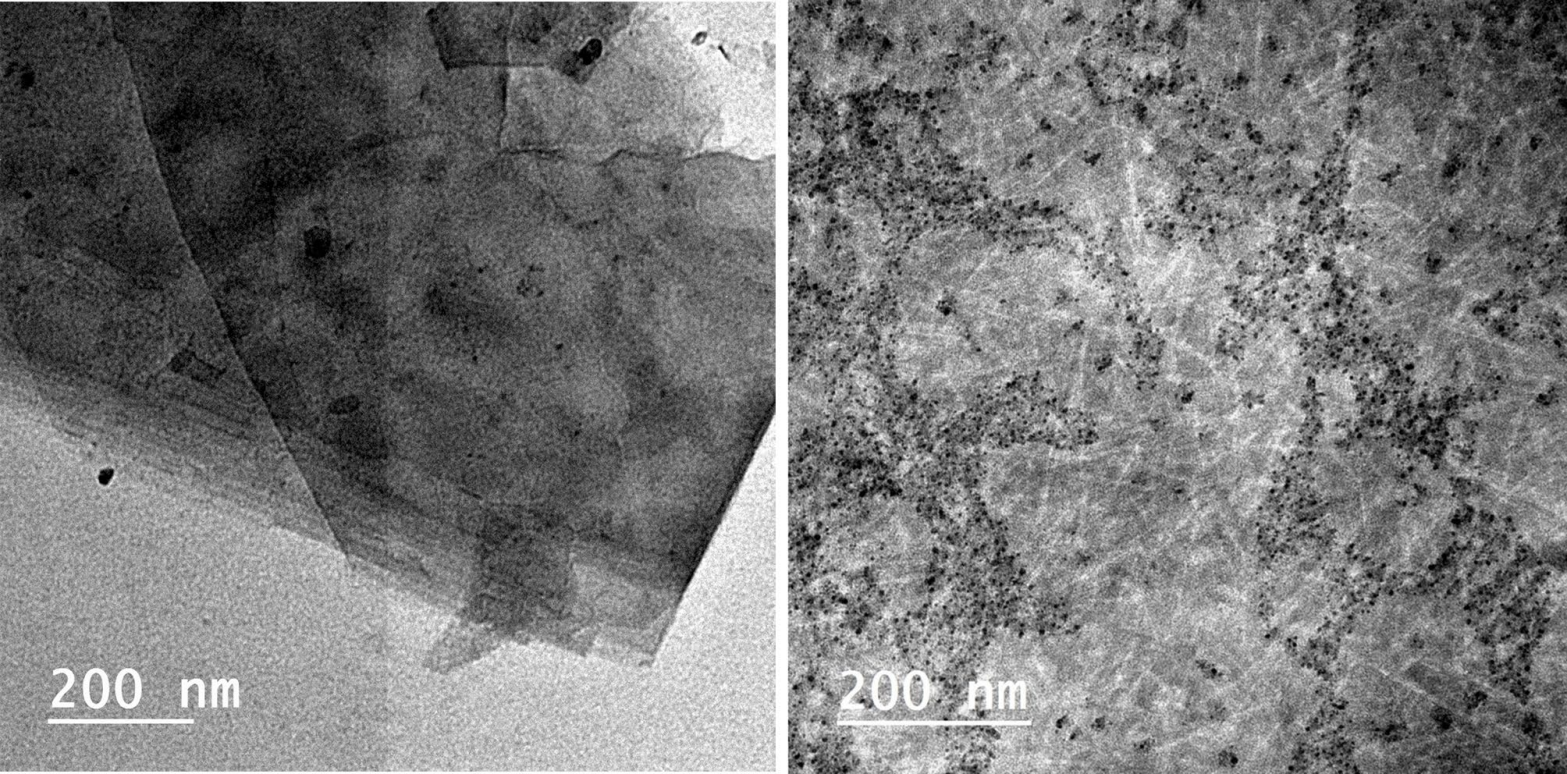
TEM of GO and CMGO

From TEM analysis, Fig. 3, we can see that GO appeared as fluffy sheets while CMGO appeared as a disintegrated structure due to carboxymethylation process.

### Biochemical analysis

Table 2 showed that there was an increase in the body weight for groups 3 and 4, in contrary to group 2, which show a decrease in body weight. Feeding rats with CCl_4_ dose, group 2, causes an increase in the liver and kidney weight% by 39.8 and 18.8%, respectively. On other hand, CMGO particles can accumulate in the liver and kidneys, potentially causing cells to swell and increase the liver weight% by 1.7 and 16.7% and the kidney weight% by 10 and 17.5% for groups 3 and 4, respectively. This can lead to an observable increase in organ weight without necessarily indicating damage or dysfunction.

In order to assess the degree of liver dysfunction in each group AST and ALT enzyme markers levels were recorded. Table 3 shows a high degree of liver dysfunction for group 2 compared to the control, group1. The percentage protection in AST marker enzyme of groups 3 was 16.67% when compared to the hepatotoxic group 2. While, group 4 showed liver dysfunction. SC juice can negatively impact liver function when combined with CMGO. An ALT activity level in blood is a very specific and sensitive clinical biomarker and preclinical of hepatotoxicity or cytotoxicity [23]. On contrary, ALT was increased for groups 3 but decreased by 2.57% for group 4, compared to the hepatotoxic group 2, which means that the treatment may be improving liver function, but there is still some ongoing damage to the liver cells.

**Table 2:** Body weight gain, food intake, liver weight % and kidney weight %

Groups	Body weight	Food intake	Liver weight (%)	Kidney weight (%)
Group 1 (Control)	9.47 ± 2.01^a^	328.00 ± 0.15^a^	2.94 ± 0.17^a^	0.80 ± 0.03^a^
Group 2	5.57 ± 1.13^a^	327.14 ± 0.18^a^	4.11 ± 0.28^b^	0.95 ± 0.05^b^
Group 3	10.60 ± 2.52^a^	327.50 ± 0.51^a^	2.99 ± 0.23^a^	0.88 ± 0.02^ab^
Group 4	12.77 ± 3.19^a^	326.86 ± 0.66^a^	3.43 ± 0.25^ab^	0.94 ± 0.0^b^

*Group 1* control negative group (basal diet), *Group 2* control positive group (basal diet + 0.4 µ CCl_4_ orally), *Group 3* control positive (group basal diet + 0.4 µ CCl_4_ orally + 1 g CMGO dissolved in 10 ml water), *Group 4* control positive group + (group basal diet + 0.4 µ CCl_4_ orally + 1 g CMGO dissolved in 10 ml sugar cane juice). a, b and c are signs for grade of differences

Compared with group 2, the urea was decreased by 7.95 and 4.26% for groups 3 and 4 respectively. In addition, uric acid was decreased by 8.79 and 11.71% for groups 3 and 4, respectively, which show the effectiveness of CMGO on the improving of the overall function of the liver, allowing it to process urea and uric acid more efficiently.

**Table 3:** Alanine aminotransferase (ALT) and aspartate aminotransferase (AST)

Groups	ALT (U/L)	AST (U/L)	Urea (mg/dl)	Uric acid (mg/dl)
Group 1 (Control)	74.06 ± 6.98^a^	83.97 ± 2.29^a^	34.23 ± 1.35^a^	4.02 ± 0.25^b^
Group 2	161.53 ± 6.84^b^	168.02 ± 13.33^c^	41.23 ± 1.76^b^	3.33 ± 0.18^a^
Group 3	187.35 ± 7.75^c^	140.00 ± 1.09^b^	37.95 ± 0.93^ab^	3.04 ± 0.15^a^
Group 4	157.37 ± 5.54^b^	170.00 ± 1.09^c^	39.47 ± 1.26^b^	2.94 ± 0.20^a^

*Group 1* control negative group (basal diet), *Group 2* control positive group (basal diet + 0.4 µ CCl_4_ orally), *Group 3* control positive (group basal diet + 0.4 µ CCl_4_ orally + 1 g CMGO dissolved in 10 ml water), *Group 4* control positive group + (group basal diet + 0.4 µ CCl_4_ orally + 1 g CMGO dissolved in 10 ml sugar cane juice). a, b and c are signs for grade of differences

### Histopathological analysis

**Fig. 4:**
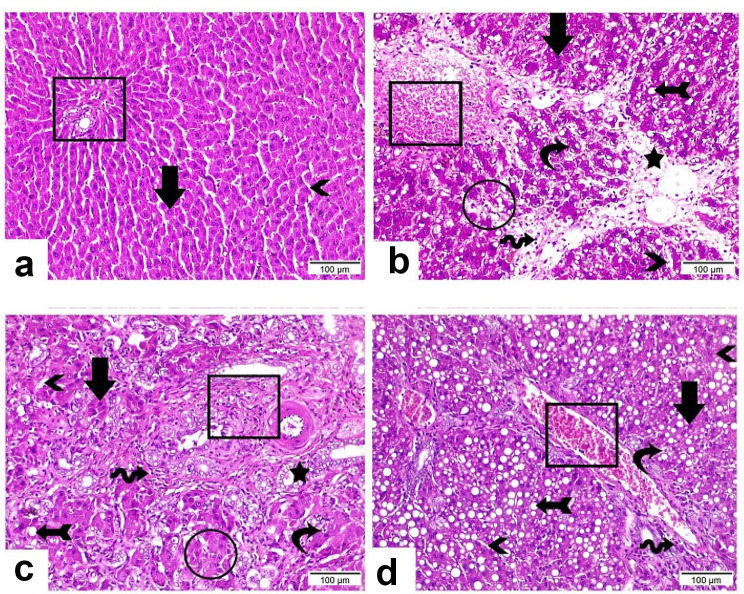
Representative photographs of histopathological changes showing effect of the CMGO material on the rats intoxicatedwith CCl_4_

A section of the liver from the control negative group 1, Fig. 4a, shows the intact blood sinusoids (arrowhead), the typical architecture of the portal area (rectangle), and the hepatic cords holding the hepatocytes with the central, spherical, and vesicular nuclei (arrows). The Liver Section of the Control Positive Group 2, Fig. 4b, demonstrated severe hepatic injury, as evidenced by the following: loss of hepatic cord organization (circle), severe dilatation and congestion of the portal vein with degenerated endothelium (rectangle), interstitial edema and fibrosis leading to dispersion between hepatic cords (star), and infiltrations of inflammatory cells (wave arrow) [24]. Additionally, certain hepatocytes were found to have deep basophilic apoptotic nucleus and deep acidophilic cytoplasm (arrow), whereas other hepatocytes had hydropic degenerations (curvy arrow). Visible dilatation was revealed by blood sinusoids (arrowhead). Take note of the tail-like micro vesicular steatosis (arrow). The effect of CMGO was obvious on group 3 and 4 (Fig. 4c and d, respectively). The liver Section from group 3 revealing a marked hepatocellular degeneration along the tissue architecture manifested by obvious increase in fibrosis (rectangle), bile duct hyperplasia (star), high number of inflammatory cells (wave arrow), complete loss of hepatic organization (circle), hepatocyte existed either with deep acidophilic cytoplasm and apoptotic nucleus (arrow) or with hydropic degeneration (curvy arrow), dilated blood sinusoids (arrowhead), as well as moderate micro vesicular steatosis (arrow with tail) [25].

In addition, the liver section from group 4 displaying less injury evidenced by moderate dilatation and congested portal vein (rectangle), less fibrosis with few numbers of inflammatory cells (wave arrow) [26]. Most hepatocytes presented with deep acidophilic cytoplasm and intact nucleus (arrow) while few hepatocytes existed with hydropic degeneration (curvy arrow). Blood sinusoids disclosed some with regular structure and others with clear atrophy (arrowheads). Moreover, severe micro vesicular steatosis was observed (arrow with tail).

## Conclusions

Novel, economical, fast and eco-friendly microwave method was employed to fabricate CMGO from SC residues. FTIR characterization confirmed the successful synthesis of CMGO by demonstrating the presence of two peaks at 1567.93 and 1639.29 cm^−1^ for CMGO, corresponding to anti-symmetric and symmetric COONa vibrations, characteristic of carboxymethylation. In addition, the relative intensity of the CH_2_ group increases from 0.60 in GO to 1.12 in CMGO, further supporting the introduction of carboxymethyl groups.

Bioactive impact of CMGO prepared from SC decrease the side effects of toxic materials such as CCL_4_. From the results of biochemical parameters of the animals it was found that both group 3 and 4 treated with CMGO have partially reduced the elevated levels of ALT, AST, urea and uric acid which means that the treatment may be improving liver function, but there is still some ongoing damage to the liver cells.

## Data Availability

All data generated or analysed during this study are included in this published article [and its supplementary information files].
